# Combination of XEOL, TR-XEOL and HB-T interferometer at the TPS 23A X-ray nanoprobe for exploring quantum materials

**DOI:** 10.1107/S1600577523010469

**Published:** 2024-01-19

**Authors:** Tzu-Chi Huang, Shang-Wei Ke, Yu-Hao Wu, En-Rui Wang, Wei-Lon Wei, Chien-Yu Lee, Bo-Yi Chen, Gung-Chian Yin, Han-Wei Chang, Mau-Tsu Tang, Bi-Hsuan Lin

**Affiliations:** a Department of Chemical Engineering, National United University, Miaoli 360302, Taiwan; b National Synchrotron Radiation Research Center, Hsinchu 300092, Taiwan; c Department of Materials Science and Engineering, National Yang Ming Chiao Tung University, Hsinchu 30010, Taiwan; Bhabha Atomic Research Centre, India

**Keywords:** X-ray nanoprobe, XEOL, TR-XEOL, HB-T

## Abstract

In this study, a combination of X-ray excited optical luminescence (XEOL), time-resolved XEOL (TR-XEOL) and the Hanbury-Brown and Twiss (HB-T) interferometer at the Taiwan Photon Source (TPS) 23A X-ray nanoprobe beamline for exploring quantum materials is demonstrated. On the basis of the excellent spatial resolution rendered using a nano-focused beam, emission distributions of artificial micro-diamonds can be obtained by XEOL maps, and featured emission peaks of a selected local area can be obtained by XEOL spectra. The hybrid bunch mode of the TPS not only provides a sufficiently high peak power density for experiments at each beamline but also permits high-quality temporal domain (∼200 ns) measurements for investigating luminescence dynamics. From TR-XEOL measurements, the decay lifetime of micro-diamonds is determined to be approximately 16 ns. Furthermore, the XEOL spectra of artificial micro-diamonds can be investigated by the HB-T interferometer to identify properties of single-photon sources. The unprecedented strategy of combining XEOL, TR-XEOL and the HB-T interferometer at the X-ray nanoprobe beamline will open new avenues with significant characterization abilities for unraveling the emission mechanisms of single-photon sources for quantum materials.

## Abstract 2

The unprecedented strategy of combining XEOL, TR-XEOL and the HB-T interferometer at the TPS 23A X-ray nanoprobe beamline will open new avenues for exploring quantum materials.

## 1. Introduction

Thomas & Senellart (2021 ▸) published a paper titled ‘*The race for the ideal single-photon source is on*’. Exploring a highly efficient and perfectly controlled single-photon source is one of the important issues in the development of quantum technologies. The color or defect centers in bulk or nano-crystals are one of the promising types of quantum emitters (Rodt *et al.*, 2020 ▸). For instance, color centers in diamond (Iwasaki *et al.*, 2015 ▸), color centers in compound semiconductors such as SiC (Castelletto *et al.*, 2014 ▸) and ZnO (Choi *et al.*, 2014 ▸), and rare-earth-ion impurities in yttrium aluminium garnet (YAG, Y_3_Al_5_O_12_) (Kolesov *et al.*, 2012 ▸) are optimal candidates for developing single-photon sources. In particular, Ko *et al.* (2007 ▸) reported that the color center can be produced by irradiating calcium oxide with soft X-rays from a synchrotron radiation source. In addition, synchrotron-based methods with high spatial resolution will provide new avenues to explore quantum materials. Plass *et al.* (2023 ▸) have reported the advantages of using a hard X-ray nanoprobe beamline to investigate Co-doped ZnO nanowires. X-ray fluorescence (XRF) can be employed to determine the Co elemental distribution, and emission properties can be measured by X-ray excited optical luminescence (XEOL). Moreover, luminescence dynamics can be investigated by time-resolved XEOL (TR-XEOL) using a streak camera. In addition to XRF, XEOL and TR-XEOL, X-ray absorption spectroscopy (XAS) is another featured method at the X-ray nanoprobe beamline. For instance, by using XAS at the X-ray nanoprobe beamline, the rare-earth valence state of the local area in rare-earth-doped samples can be clearly observed (Wu *et al.*, 2022 ▸).

The X-ray nanoprobe beamlines at synchrotron facilities include ID16A (Villar *et al.*, 2018 ▸) and ID16B (Martínez-Criado *et al.*, 2016 ▸) at the European Synchrotron Radiation Facility, the HERMES beamline of Synchrotron SOLEIL (Hageraats *et al.*, 2021 ▸), 26-ID-C at the Advanced Photon Source (Winarski *et al.*, 2012 ▸), I14 at Diamond Light Source (Quinn *et al.*, 2021 ▸), NanoMAX at MAX IV (Johansson *et al.*, 2021 ▸), CARNAÚBA of the Laboratório Nacional de Luz Síncrotron (Tolentino *et al.*, 2017 ▸), PETRA III beamline P06 of the Deutsches Elektronen-Synchrotron (Schroer *et al.*, 2016 ▸), 3-ID HXN beamline of the National Synchrotron Light Source II (Nazaretski *et al.*, 2017 ▸), BL37XU and BL39XU beamlines at SPring-8 (Koyama *et al.*, 2011 ▸) and the TPS 23A beamline at the Taiwan Photon Source (TPS) (Lin *et al.*, 2020 ▸; Wu *et al.*, 2022 ▸). The TPS 23A X-nanoprobe not only exhibits proficiency in XEOL, TR-XEOL, XAS and XRF measurements but also comprises an HB-T interferometer built into the endstation. The HB-T interferometer (Hanbury-Brown & Twiss, 1979 ▸; Glauber, 1963*a* ▸; Hanbury-Brown & Twiss, 1956 ▸) can be employed to investigate the emission properties of quantum materials whether or not these materials are single-photon sources. The advantages of XEOL and TR-XEOL have been clearly reported by Martínez–Criado *et al.* (2014 ▸) and Armelao *et al.* (2010 ▸) in the hard and soft X-ray fields, respectively. The inclusion of the HB-T interferometer with XEOL and TR-XEOL is hypothesized to lead to tremendous progress in the capabilities of the X-ray nanoprobe.

In this study, artificial micro-diamonds are used to demonstrate the capabilities and advantages of using XEOL, TR-XEOL and the HB-T interferometer. According to the excellent spatial resolution using a nano-focused beam, the emission spectra of an artificial micro-diamond with different local areas can be investigated by XEOL. The XEOL maps with different emission wavelengths can be used to further determine the emission intensity distributions. The TPS operating in the hybrid bunch mode not only provides a sufficiently high peak power density for each beamline but also permits high-quality temporal domain measurements for investigating the luminescence dynamics of materials. The decay lifetime of an artificial micro-diamond with different local areas can be investigated by TR-XEOL. Finally, the XEOL emission intensities of an artificial micro-diamond with different local areas are measured using the HB-T interferometer to analyze properties of the second-order correlation function *g* ^(2)^(τ).

## 2. Experiment

**Figure 1:**
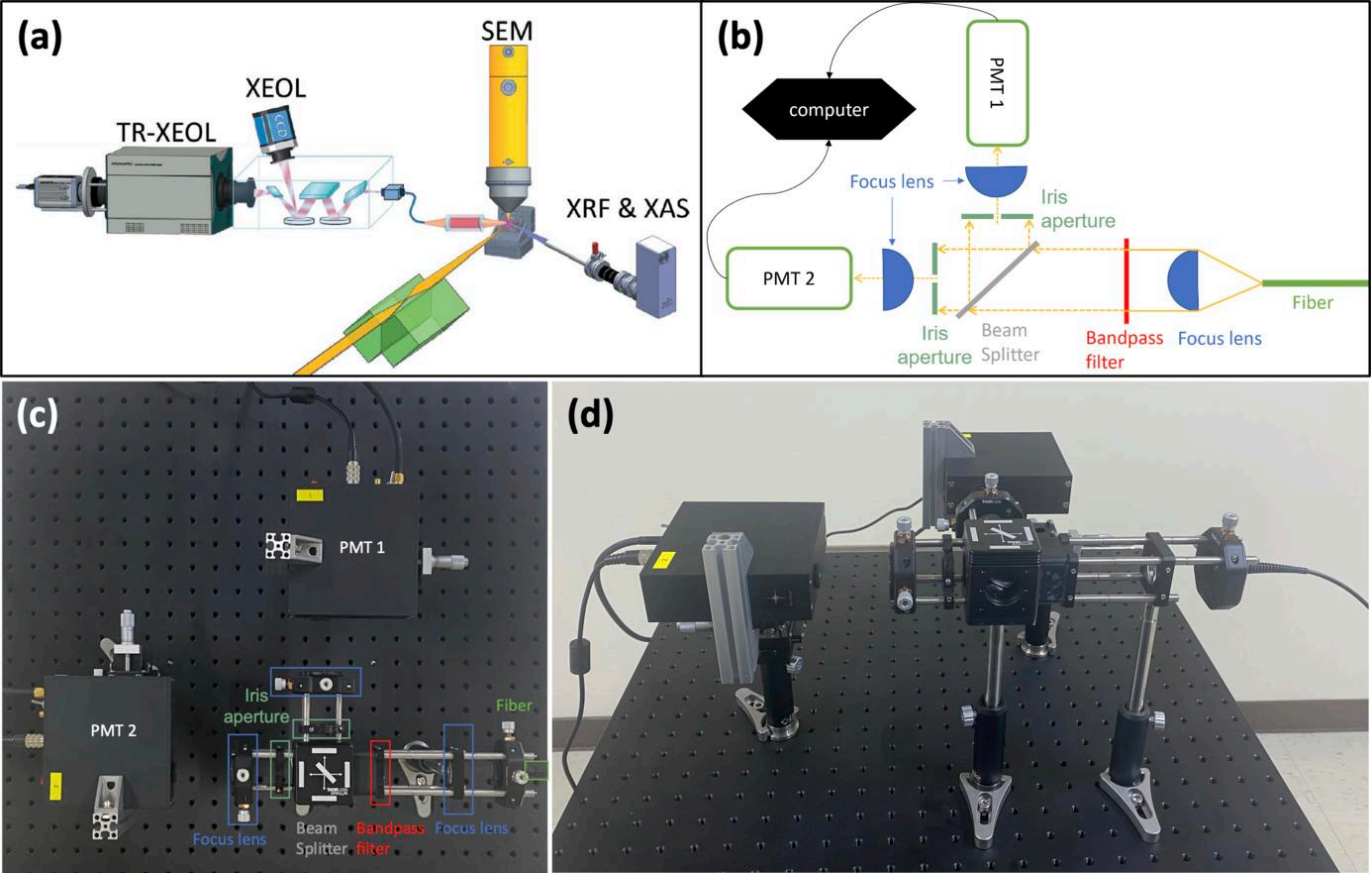
(*a*) Multi-functions of the TPS 23A X-ray nanoprobe, including XEOL, TR-XEOL, XRF, XAS and SEM. (*b*) Detailed optical path of the HB-T interferometer, which is new equipment installed at TPS 23A. Panels (*c*) and (*d*) show top- and side-view photographs of the HB-T interferometer, respectively. The diagram in panel (*a*) is adapted from Lin *et al.* (2020 ▸).

Artificial micro-diamonds, purchased from FND BIotech, Inc., are used as the test samples. Fig. 1 ▸(*a*) shows the multi-function methods of TPS 23A, including XEOL, TR-XEOL, XAS and XRF. A detailed description has been reported by Lin *et al.* (2020 ▸). By using an optical fiber attached to a spectrometer (iHR550, Horiba), we collected XEOL spectra and XEOL maps by using a deep thermoelectric cooling charge-coupled device (Syncerity BI UV-Vis with 2048 × 70 pixels) and a photomultiplier tube (PMT), respectively. TR-XEOL can be measured by switching the optical fiber to another spectrometer (iHR320, Horiba), which is equipped with a Hamamatsu C10910 streak camera and an M10913 slow single-sweep unit. XRF and XAS measurements can be conducted using a silicon drift detector (Vortex-ME4, Hitachi). Fig. 1 ▸(*b*) shows the detailed optical path of the HB-T interferometer, which is new equipment installed on TPS 23A. Figs. 1 ▸(*c*) and 1 ▸(*d*) show top- and side-view photographs of the HB-T interferometer, respectively. In addition, by switching the optical fiber, the XEOL intensity can be directed into the HB-T interferometer, and the measured emission wavelength can be selected by using a bandpass filter. The emission intensity of the selected wavelength can be measured by PMT1 and PMT2 (NIR1, Southport) through a beam splitter (50/50 ratio). Finally, the signals of both PMTs are analyzed by using the MultiHarp 150 module (PicoQuant) and *SymPhoTime 64-1* software (PicoQuant) to obtain the second-order correlation function *g* ^(2)^(τ).

XEOL, TR-XEOL and HB-T can be switched easily and rapidly through the optical fiber.

## 3. Results and discussion

### 3.1. XEOL map and XEOL spectra

**Figure 2:**
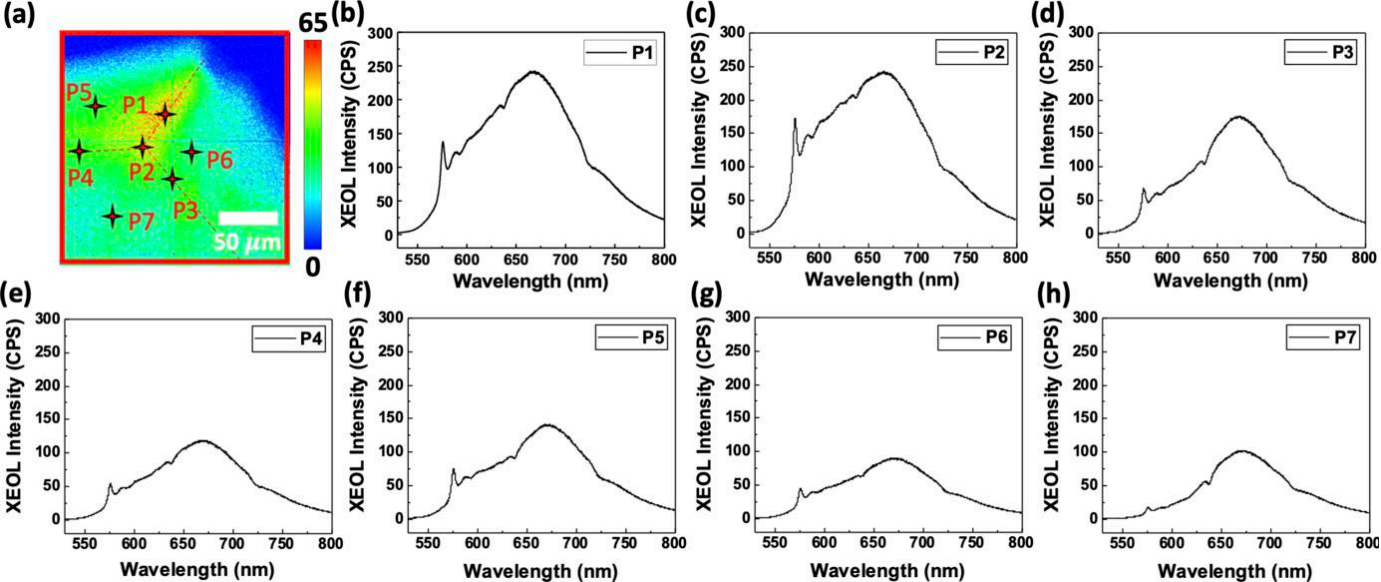
(*a*) Emission intensity distribution of the artificial micro-diamond with λ_em_ = 665 nm. XEOL emission spectra of the local areas of P1–P7 (*a*) are shown in (*b*)–(*h*), respectively.

The two charge states in the nitro­gen-vacancy (NV) centers of micro-diamond are NV^0^ (neutral) and NV^−^ (negatively charged) (Doherty *et al.*, 2013 ▸). The sharp emission peaks of the zero photon line (ZPL) of NV^0^ and NV^−^ are located at 575 nm and 638 nm, respectively (Lu *et al.*, 2019 ▸). Fig. 2 ▸(*a*) shows the emission intensity distribution of the artificial micro-diamond with wavelength λ_em_ = 665 nm. As can be observed clearly from the XEOL map, the emission intensity of λ_em_ = 665 nm is not uniform. On the basis of the excellent spatial resolution using the nano-focused beam, different local areas P1–P7 marked in Fig. 2 ▸(*a*) are selected to investigate their XEOL emission spectra [Figs. 2 ▸(*b*)–2(*h*)]. The XEOL spectra of P1–P7 reveal similar emission behaviors: the ZPL of NV^0^ is observable at 575 nm, and the board phonon side band (Schreyvogel *et al.*, 2015 ▸) ranges from 600 nm to 750 nm. The ZPL of NV^0^ at P1 and P2 exhibits the strongest emission intensity, while that at P7 exhibits the lowest emission intensity. The results imply that NV aggregates at the corners and edges of the diamond. Compared with the sharp and clear peak of the ZPL of NV^0^ at 575 nm, the ZPL of NV^−^ at 638 nm reveals an extremely weak emission intensity. From the XEOL spectra, the emission properties of the artificial micro-diamond can be determined rapidly. In addition to our studies, Zhou *et al.* (2009 ▸) have also shown the advantages of using XEOL to investigate the emission mechanism of the carbon nanocrystals, natural diamond and CVD nanodiamond.

**Figure 3:**
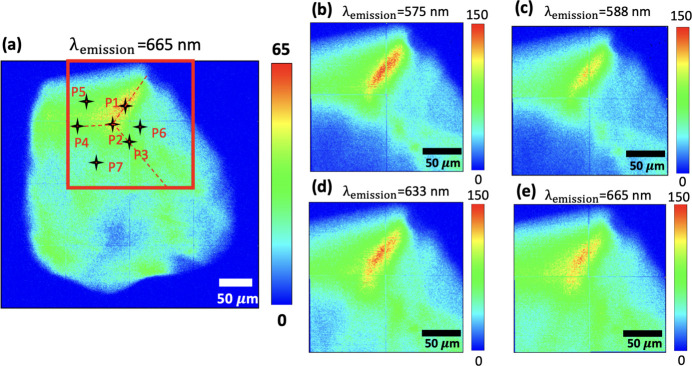
(*a*) Magnified image of the artificial micro-diamond at λ_em_ = 665 nm. Panels (*b*), (*c*), (*d*) and (*e*) show emission distributions with λ_em_ = 575, 588, 633 and 665 nm, respectively, representing the area marked by the red square in (*a*).

Fig. 3 ▸(*a*) shows the full size of the artificial micro-diamond with λ_em_ = 665 nm, where the XEOL map reveals that the size of the artificial micro-diamond is approximately 300 µm. To further understand the emission distribution under different emission wavelengths, four emission wavelengths were selected to plot the emission distributions in the area marked by the red square shown in Fig. 3 ▸(*a*). Figs. 3 ▸(*b*), 3 ▸(*c*), 3 ▸(*d*) and 3(*e*) show the emission distributions with λ_em_ = 575, 588, 633 and 665 nm, respectively. The XEOL map shown in Fig. 3 ▸(*b*) clearly reveals the emission distribution of the ZPL of NV^0^ at 575 nm; this result confirms our hypothesis that NV aggregates at the corners and edges of the diamond.

### 3.2. TR-XEOL

**Figure 4:**
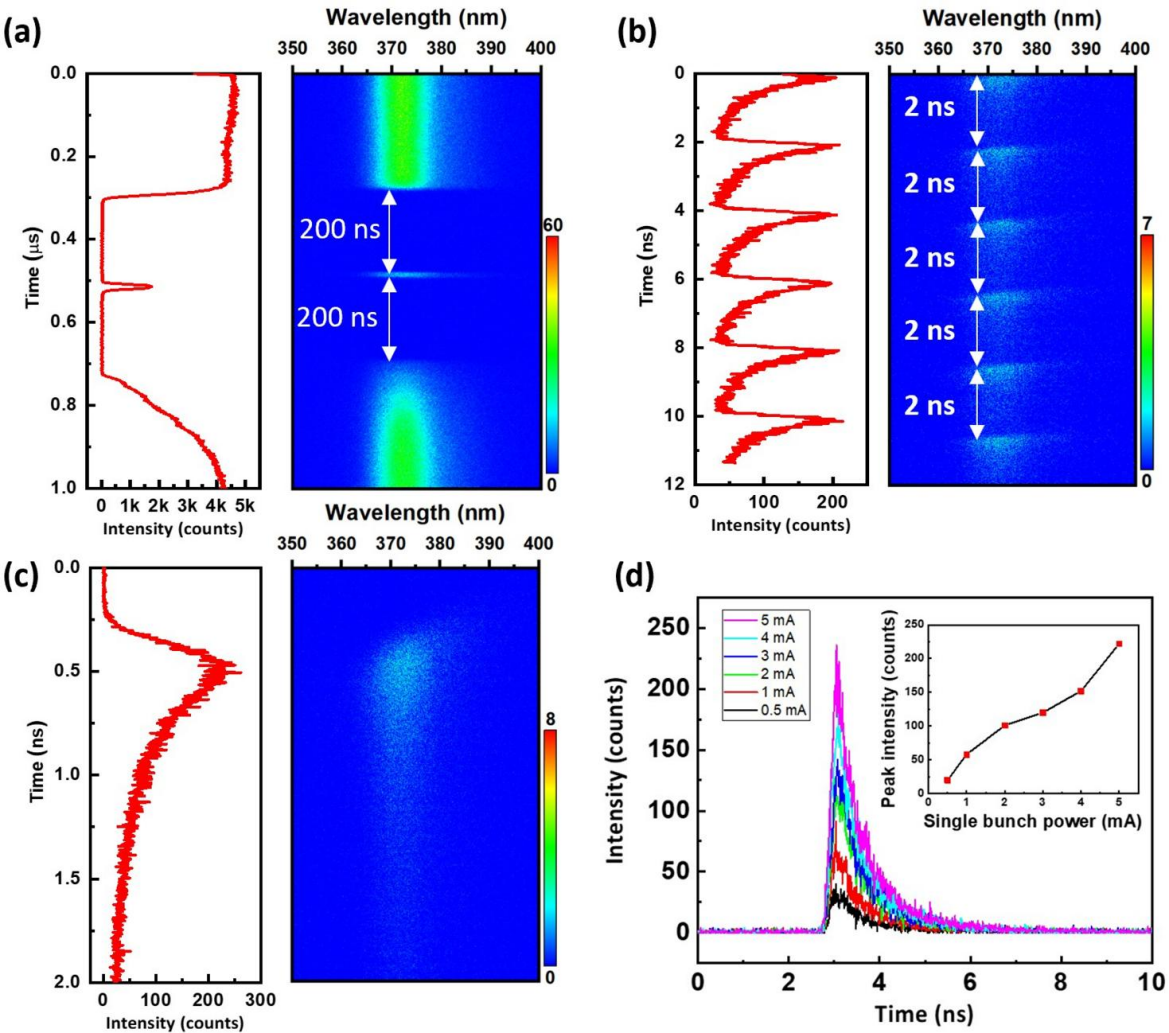
The right-hand plots in panels (*a*), (*b*) and (*c*) show streak images using time windows of 1 µs, 12 ns and 2 ns, respectively; the left-hand plots represent the spectral integrated (366–376 nm) time traces of each streak image. (*d*) TR-XEOL spectra of a single bunch with different beam currents. The inset of (*d*) reveals that the emission peak intensity of TR-XEOL increases with an increase in the beam current.

TPS 23A comprises not only XEOL to investigate the emission wavelength and distribution of the sample but also TR-XEOL to investigate luminescence dynamics. The advantages of TR-XEOL have also been reported by Ward *et al.* (2021 ▸) using the Canadian Light Source. Concerning the resolution of the TR-XEOL, we should consider the instrument response function (IRF). The IRF should take into account three main factors – the pulsed duration times of the source (τ_s_), the electronics (τ_e_) and the detector (τ_d_), and can be approximated to IRF ≅ [(τ_s_)^2^ + (τ_e_)^2^ + (τ_d_)^2^]^1/2^. Then we can calculate IRF ≅ 45 ps using τ_s_ ≅ 30 ps, τ_e_ ≅ 25 ps and τ_d_ ≅ 24 ps in the case of a streak image with 2 ns sweep time at TPS 23A (Lin *et al.*, 2020 ▸). Three different filling patterns of the electron bunches can be used at the TPS: the single, multi- and hybrid bunch modes, which provide time scales from 45 ps to 1.72 µs, 45 ps to 2 ns and 45 ps to 200 ns, respectively. A *c*-plane GaN wafer is used as the standard sample to identify the filling pattern of the electron bunches in the TPS storage ring. As the decay lifetime of the near-band-edge emission (∼373 nm) of GaN is approximately ∼385 ps, the emission intensity of GaN can be measured to demonstrate that the TPS is operating in the hybrid bunch mode. Fig. 4 ▸(*a*) shows a streak image using TR-XEOL to measure the *c*-plane GaN wafer. To render a higher emission intensity, the X-ray energy is tuned at 10.375 keV, which is greater than that of the Ga *K*-edge (10.367 keV) and at the resonance point of the Ga absorption spectrum. The streak image clearly demonstrates that TPS is operating in the hybrid bunch mode, which is attributed to the clearly observed emission intensity from multi-bunches and a single bunch, especially in the case of the 200 ns time span between the multi-bunches and single bunch. As the streak image of the 1 µs time window cannot resolve the multi-bunches, the multi-bunches with a 2 ns time span can be observed clearly by tuning the time window to 12 ns [Fig. 4 ▸(*b*)]. By the same reasoning, more suitable time resolution can be achieved by tuning the time window to 2 ns to measure the decay behavior of the GaN from the single bunch [Fig. 4 ▸(*c*)]. Furthermore, the beam current of the single bunch can be tuned from 0.5 mA to 5 mA, and the corresponding TR-XEOL spectra results are shown in Fig. 4 ▸(*d*). The inset of Fig. 4 ▸(*d*) reveals that the emission peak intensity of TR-XEOL increases with the increase in the beam current. After several experiments, the most appropriate and stable beam current of the single bunch is 3.5 mA, which is approximately six times greater than that of the multi-bunches. The hybrid bunch mode is advantageous as it provides a sufficiently high peak power density for each beamline in TPS and permits high-quality temporal domain measurements for investigating the luminescence dynamics of materials.

**Figure 5:**
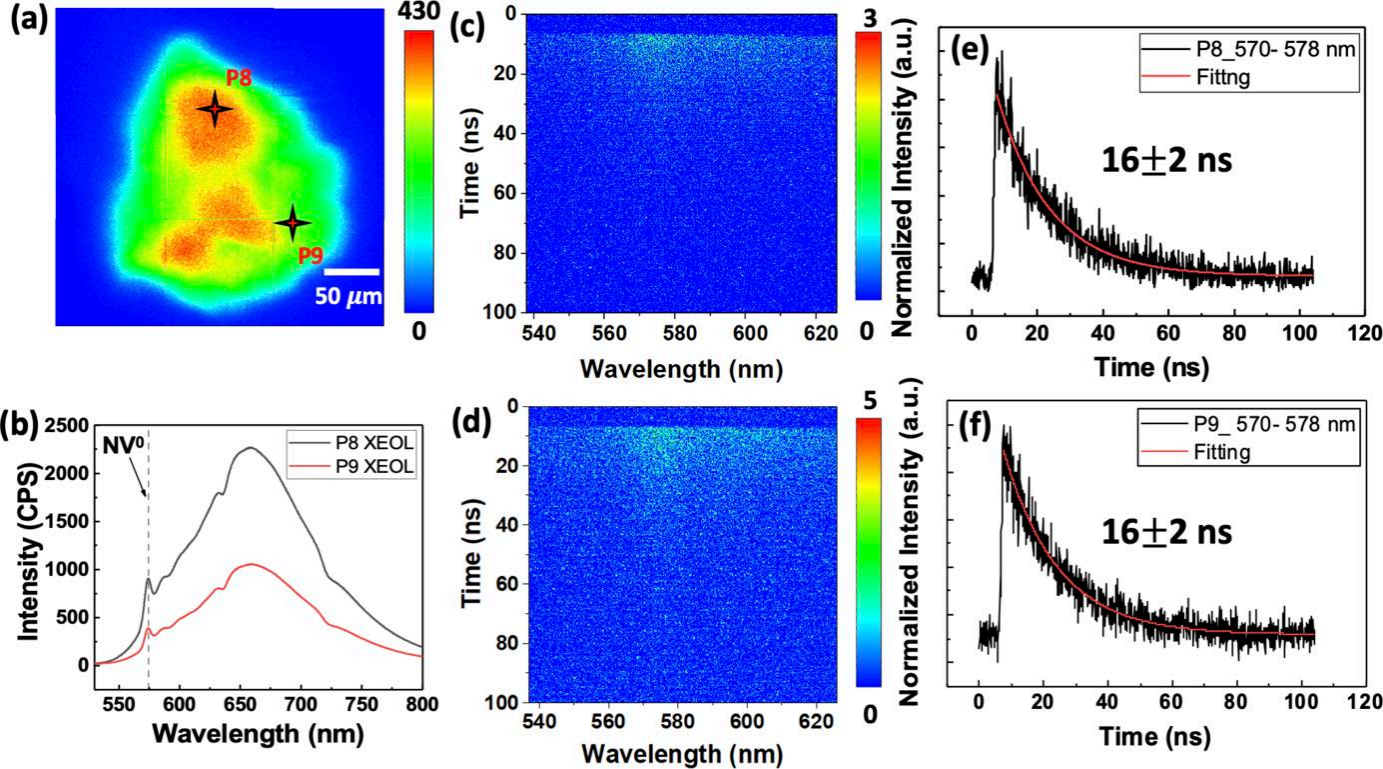
(*a*) XEOL map with λ_em_ = 575 nm. (*b*) XEOL spectra of areas P8 and P9 marked in (*a*). Streak image (*c*) and spectral integrated (570–578 nm) time traces (*e*) of P8, as well as the corresponding data for P9 shown in (*d*) and (*f*).

Another artificial micro-diamond was selected to confirm the capabilities of TR-XEOL. The emission distribution of the micro-diamond with λ_em_ = 575 nm can be obtained easily using the XEOL map [Fig. 5 ▸(*a*)]. Two local areas with higher (P8) and lower 575 nm emission intensities were selected to measure XEOL spectra [Fig. 5 ▸(*b*)]. Figs. 5 ▸(*c*) and 5 ▸(*d*) show the streak images of P8 and P9, respectively, using TR-XEOL. By using one exponential decay function to fit the spectral integrated (570–578 nm) time traces shown in Figs. 5 ▸(*c*) and 5 ▸(*d*), the decay lifetimes of P8 and P9 are found to be 16 ± 2 ns. The measured decay lifetime of P8 and P9 are consistent with that of NV in the micro-diamond by approximately 12–22 ns, which is reported by Aharonovich *et al.* (2016 ▸). The results also indicate that the emission intensities of the ZPL of NV^0^ at 575 nm do not affect the decay lifetime.

### 3.3. HB-T interferometer

In our previous studies, we found some peculiar emission behaviors when using an X-ray nanoprobe with XEOL and TR-XEOL. For instance, the emission intensity of the non-polar *a*-plane MgZnO/ZnO multiple quantum wells will increase by more than ten times after high X-ray irradiation (Lin *et al.*, 2019 ▸). We used XEOL to investigate the emission properties of MgAl_2_O_4_ wafers, and then identified 13 and 6 clear emission peaks corresponding to the color centers in MgAl_2_O_4_ wafers and the ^2^ *Eg* → ^4^ *A*_2*g*_ radiative transition of Cr ion impurities, respectively. In addition, Ko *et al.* (2007 ▸) also used XEOL and TR-XEOL to observe the *F* and *F* ^+^ color centers of CaO powder. They found that the emission intensity of the *F* center in CaO will also increase with the irradiation time, which indicates that a color center can be produced by irradiating X-rays using a synchrotron radiation source. Generating color centers effectively in wide-band-gap materials is important to the realization of solid state quantum technologies (Smith *et al.*, 2019 ▸). Using an X-ray nanoprobe with a synchrotron radiation source can provide not only the generation but also the positioning of color centers in wide-band-gap materials. So, combining the HB-T interferometer with an X-ray nanoprobe will open new avenues with significant characterization abilities for exploring quantum materials.

One of the most important characteristic functions for a light source is the second-order correlation function *g* ^(2)^ (Plenio & Knight, 1998 ▸). The non-classical anti-bunching light sources can be distinguished from the classical thermal ones by using *g* ^(2)^ (Huang *et al.*, 2016 ▸). The HB-T interferometer of Fig. 1 ▸(*c*) is composed of two detectors, which can detect the correlation of incident light intensity from the beam splitter. τ, of *g* ^(2)^(τ), represents the time difference between the photons detected by the two detectors. For instance, *g* ^(2)^(τ = 0) can be regarded as the possibility that both detectors detect photons at the same time. An optical interferometer was set up by R. Hanbury-Brown and R. Q. Twiss, called the HB-T interferometer (Hanbury-Brown & Twiss, 1956 ▸). Glauber (1963*b* ▸) developed quantum mechanical methods for the photon correlations to explain the bunching effect observed by HB-T. Then, the phenomenon of photon anti-bunching was observed by Kimble *et al.* (1977 ▸). Thus, the HB-T interferometer and second-order correlation function *g* ^(2)^ can be used to study single-photon sources.

**Figure 6:**
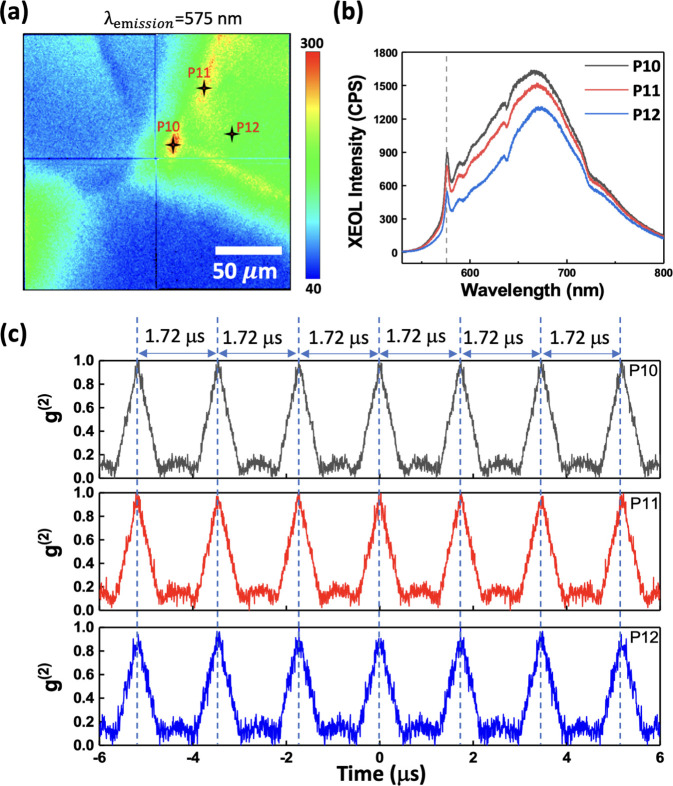
(*a*) XEOL map with λ_em_ = 575 nm. (*b*) XEOL spectra of P10, P11 and P12 marked in (*a*). (*c*) Second-order correlation function *g* ^(2)^(τ) of P10, P11 and P12.

In addition to XEOL and TR-XEOL, the emission properties of the artificial micro-diamond can be investigated using the HB-T interferometer. As described in the previous section, the emission distribution (λ_em_ = 575 nm) and spectra can be measured easily by using the XEOL map and XEOL spectra, respectively [Figs. 6 ▸(*a*) and 6 ▸(*b*)]. The emission intensities of P10, P11 and P12 are selected with the HB-T interferometer to analyze their second-order correlation function *g* ^(2)^(τ). The 575 nm bandpass filter with a bandwidth of 27 nm was used to filter out the other emission peaks, and only the emission peak of the ZPL of NV^0^ at 575 nm was allowed to pass into the HB-T interferometer. Fig. 6 ▸(*c*) shows *g* ^(2)^(τ) of P10, P11 and P12. As the repetition rate of the single bunch in the TPS is 578 kHz (period of 1.72 µs), Fig. 6 ▸(*c*) shows clearly that the time span of each peak is 1.72 µs. These results demonstrate that the HB-T interferometer is synchronized successfully with the TPS 23A beamline using the single-bunch mode. Although the *g* ^(2)^(0) values of P10, P11 and P12 are approximately 1, indicating that the emission light of the ZPL of NV^0^ at 575 nm is not a single-photon source, the HB-T interferometer is successfully combined into TPS 23A. This indicates that the TPS 23A X-ray nanoprobe is capable of investigating the bunching or anti-bunching properties of quantum materials to explore single-photon sources.

## 4. Conclusions

In this study, the XEOL map, XEOL spectra, TR-XEOL and the HB-T interferometer at the TPS 23A X-ray nanoprobe beamline were successfully used to investigate the emission distributions, emission properties, decay lifetimes and second-order correlation function *g* ^(2)^(τ) of artificial micro-diamonds. The nano-focused beam and operation in the hybrid bunch mode can render the advantages of excellent spatial resolution (<100 nm) as well as a sufficiently high peak power density for each beamline at TPS. In addition, high-quality temporal domain measurements for investigating the luminescence dynamics of materials are possible. The XEOL map and XEOL spectra reveal that the nitro­gen vacancy aggregates at the corners and edges of the diamond. TR-XEOL results reveal that the different emission intensities of the ZPL of NV^0^ at 575 nm do not affect the decay lifetime. The combination of the HB-T interferometer with TPS 23A reveals that this beamline can investigate the properties of single-photon sources of quantum materials. The unprecedented methods of combining XEOL, TR-XEOL and the HB-T interferometer at the TPS 23A X-ray nanoprobe beamline will open new avenues with significant characterization abilities for un­raveling the emission mechanisms of single-photon sources for quantum technology.
